# Organotin Antifouling Compounds and Sex-Steroid Nuclear Receptor Perturbation: Some Structural Insights

**DOI:** 10.3390/toxics11010025

**Published:** 2022-12-27

**Authors:** Mohd A. Beg, Md A. Beg, Ummer R. Zargar, Ishfaq A. Sheikh, Osama S. Bajouh, Adel M. Abuzenadah, Mohd Rehan

**Affiliations:** 1 Reproductive Biology Laboratory, King Fahd Medical Research Center, King Abdulaziz University, Jeddah 21589, Saudi Arabia; 2 Centre for Interdisciplinary Research in Basic Sciences, Jamia Millia Islamia University, New Delhi 110025, India; 3 Department of Zoology, Government Degree College, Anantnag 192101, India; 4 Department of Medical Laboratory Technology, Faculty of Applied Medical Sciences, King Abdulaziz University, Jeddah 21589, Saudi Arabia; 5 Department of Obstetrics and Gynecology, Faculty of Medicine, King Abdulaziz University, Jeddah 21859, Saudi Arabia; 6 King Fahd Medical Research Center, King Abdulaziz University, Jeddah 21589, Saudi Arabia

**Keywords:** organotins, butyltin, phenyltin, azocyclotin, androgen receptor, estrogen receptor, molecular docking, MD simulation, endocrine disruption

## Abstract

Organotin compounds (OTCs) are a commercially important group of organometallic compounds of tin used globally as polyvinyl chloride stabilizers and marine antifouling biocides. Worldwide use of OTCs has resulted in their ubiquitous presence in ecosystems across all the continents. OTCs have metabolic and endocrine disrupting effects in marine and terrestrial organisms. Thus, harmful OTCs (tributyltin) have been banned by the International Convention on the Control of Harmful Antifouling Systems since 2008. However, continued manufacturing by non-member countries poses a substantial risk for animal and human health. In this study, structural binding of common commercial OTCs, tributyltin (TBT), dibutyltin (DBT), monobutyltin (MBT), triphenyltin (TPT), diphenyltin (DPT), monophenyltin (MPT), and azocyclotin (ACT) against sex-steroid nuclear receptors, androgen receptor (AR), and estrogen receptors (ERα, ERβ) was performed using molecular docking and MD simulation. TBT, DBT, DPT, and MPT bound deep within the binding sites of AR, ERα, and Erβ, showing good dock score, binding energy and dissociation constants that were comparable to bound native ligands, testosterone and estradiol. The stability of docking complex was shown by MD simulation of organotin/receptor complex with RMSD, RMSF, Rg, and SASA plots showing stable interaction, low deviation, and compactness of the complex. A high commonality (50–100%) of interacting residues of ERα and ERβ for the docked ligands and bound native ligand (estradiol) indicated that the organotin compounds bound in the same binding site of the receptor as the native ligand. The results suggested that organotins may interfere with the natural steroid/receptor binding and perturb steroid signaling.

## 1. Introduction

Organotin compounds (OTCs), also called as stannanes, are a large class of organometallic compounds having at least one tin atom covalently bound to a carbon atom with a general formula ‘RSnX’ where ‘R’ represents an organic group, i.e., alkyl, phenyl, etc., and ‘X’ an anion such as chloride, fluoride, oxide, etc. [1,2,3,4,5]. Tin metal and its alloys have been used by humans for more than 6000 years, however, the first OTC was synthesized only about 175 years back in the year 1853, and, for nearly another 100 years, OTCs were not found to have any industrial utility for want of commercial applications [5,6]. As of now, there are more than 800 OTCs known, and, barring a few, all of them are anthropogenic [7]. OTCs constitute one the largest group of organometallic compounds in commercial use globally, and the United States and China are the largest consumers of OTCs, followed by Western Europe, the Middle East, and others [8]. The downside of the increased industrial applications and use has been that considerable amounts of OTCs have entered various ecosystems, and OTCs are now ubiquitous in the environment. The worldwide higher use and demand have resulted in an increase in global production of OTCs during the last several decades. Overall, the maximum utilization of OTCs is as polyvinyl chloride (PVC) stabilizers (about 70–80% of global consumption) and remaining as biocides [5,6,9].

OTCs are classified according to the number of organic functional groups on the tin atom or the number of ‘carbon-tin’ bonds, e.g., mono-, di-, tri-, and tetra-organotin compounds [5]. Generally, mono- and di-substituted OTCs, e.g., dibutyltin (DBT), diphenyltin (DPT), etc., are used as heat stabilizers for PVC plastics, lubricating oils, hydrogen peroxide, and polyolefins and as catalysts in the production of polyurethane foam, polymers, esters, plastisol prints, rubber, adhesives, etc., with applications in apparel and footwear industries [2,5]. Tri-substituted OTCs, e.g., tributyltin (TBT), triphenyltin (TPT), etc., have been used extensively as biocides, such as antifouling paints for boats, timber preservatives, preservatives in textiles, leathers and synthetic leathers, fungicides in crops including potatoes, sugar beets, and pecans, and as pesticides [2,5,10]. OTCs are also utilized in silicone-based finishes having elastomeric and water repellency properties. The seven most common OTCs commercially available for a variety of industrial applications are TBT, DBT, monobutyltin (MBT), TPT, DPT, monophenyltin (MPT), and azocyclotin (ACT).

Environmental contamination with OTCs has been reported to be mainly through agricultural runoffs and marine aquatic environment due to leaching from antifouling paints, treated timber, PVC pipes, and other contaminated material [4,5,10]. Biodegradation half-lives of organotins are generally shorter in tri-substituted organotins compared to di-substituted organotins, whereas monosubstituted organotins have the longest half-lives. The half-lives are longer in both seawater and soil/sediment than in fresh water and can range from six months to 15 years [5]. The ‘C-Sn’ bond of organotins is comparatively stable, as the anionic group is reported to dissociate easily on hydrolysis in water. Human exposure occurs through drinking water and eating food, especially aquatic food such as fish and other marine animals. The global terrestrial and aquatic contamination and the adverse effects on non-target organisms including abnormalities in shell calcification in oysters and the masculinization of female gastropods—imposex has resulted in outlawing the use of OTs, especially tributyltin in marine paints in 2008 under the International Convention on the Control of Harmful Antifouling Systems on Ships and World Health Organization [5,10,11]. However, developing nations that are not members of the International Maritime Organization are still permitted to use OTCs for industrial and agricultural purposes. Organotins have been found in household dust in many countries, including the United States and Germany [12,13]. Due to an increase in the usage of antifouling paints containing organotin compounds, organotin contaminations have also been observed in Saudi Arabia in recent years in fishing harbors of various coastal sites in the Eastern Province (Jubail, Khobar, and Qatif) [14,15]; the estimations of the contamination have shown more phenyltins than other organotins. The detection of high concentrations of hazardous triorganotins in the commercially important fish species caught from Arabian Gulf is of particular concern for human health in Saudi Arabia [16].

Limited epidemiological studies have been reported on the exposure of humans to organotin toxicity. In the United States, MBT, DBT, and TBT were detected in 70% of the human blood samples that were tested [17]. The potential toxicological significance of OTCs to humans on the basis of animal experiments indicates diverse health problems including immunosuppression, endocrine problems, neurotoxicity, metabolic and enzymatic problems, ocular, cardiovascular, gastrointestinal, reproductive, developmental, and several other problems [18]. OTCs cause morphological and functional changes in a number of tissues in animals that are involved in the regulation of endocrine function and metabolism in mammals, including the hypothalamus, pituitary, pancreas, gonads, adipose tissue, adrenal, and thyroid glands [19,20]. In regard to reproductive problems in humans, organotin exposure has been associated with congenital abnormalities in neonates such as cryptorchidism and hormone disbalance for LH and testosterone in newborn boys [21]. Organotins have been also reported to cause metabolic dysfunction in animal models similar to polycystic ovarian syndrome (PCOS) in women [22]. In animals, organotin exposure leads to phenotypic abnormalities, aberrant changes in hormones, transcriptome and proteome modifications, behavioral changes, and characteristic sexual organ masculinization called imposex, in which female gastropods develop penile structure and vas deferens [23,24]. In rats, organotins are associated with reduced sperm viability and motility, abnormalities of spermatids and spermatozoa, testicular necrosis, changes in the blood–testicular barrier, and hormone disbalance in males [24,25,26], as well as delayed follicular development, irregular estrous cycles, alterations in steroidogenic enzymes, abnormal adipogenesis, decline in fertility, failure of implantation, lower chances of conception, and other reproductive abnormalities in females [27,28,29].

The available literature indicates that very few epidemiological or clinical studies have been reported on the effects of organotins on human health despite the overwhelming evidence of the adverse effects on laboratory and marine species. Computational methods have been increasingly used for the prediction of binding pose and molecular interactions of ligands with their target protein molecules for the characterization of interactions, designing novel inhibitors, and/or as an aid for designing experimental and clinical studies [30,31,32]. The present study was performed to characterize the structural binding interactions of seven commonly available OTCs, viz., TBT, DBT, MBT, TPT, DPT, MPT, and ACT, against sex-steroid nuclear receptors, i.e., androgen receptor (AR) and estrogen receptors (ERα, ERβ). AR and ER are soluble proteins and function as intracellular transcription factors. Testosterone and dihydrotestosterone are the main steroid ligands of AR. In addition to the development of male sexual organs, maturation of male sexual organs and spermatogenesis, AR signaling plays a crucial role in a number of physiological and developmental processes related to male physiology. ER regulates the transcription of numerous genes, and its primary role is in the development of female phenotype, development of female reproductive tract, regulation of folliculogenesis, uterine development, and in other processes related to reproductive cycle. The suggested hypothesis is that OTCs may interfere with the natural interaction between ligands and proteins by binding to sex steroid nuclear receptors in the body, which might result in dysfunction of sex steroids target organs.

## 2. Materials and Methods

### 2.1. Data Retrieval

**Figure 1:**
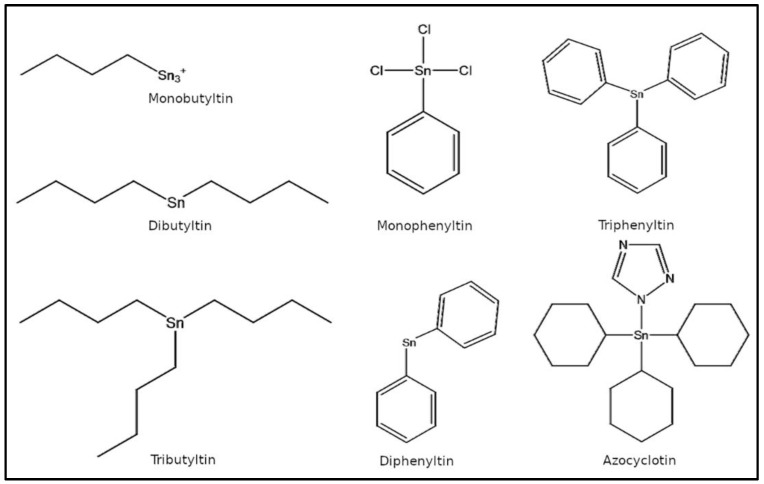
Two-dimensional structures of organotin compounds.

The three-dimensional crystal structures of the human sex-steroid hormone receptors were retrieved from the Protein Data Bank (PDB; https://www.rcsb.org, accessed on 4 December 2022) as AR (PDB ID: 2AM9), ERα (PDB ID: 5DXB), and ERβ (PDB ID: 5TOA). The two-dimensional structures of OTCs (TBT, DBT, MBT, TPT, DPT, MPT, and ACT) as illustrated (Figure 1) were retrieved from the PubChem database (https://pubchem.ncbi.nlm.nih.gov, accessed on 4 December 2022). PyMOL graphic interface was used for illustration and analysis of hormone receptors with their bound native ligands and binding pocket studies [33].

### 2.2. Molecular Docking

Molecular docking of the organotin compounds in the ligand binding sites of AR, ERα, and ERβ was carried out using Dock v.6.5 [34]. The initial structure preparation of the ligands and the receptors was performed using Chimera v.1.6.2 [35]. The native bound ligands were used as clue for the binding site and the residues around 10 Å of the native ligands were used as the binding site region for generating docking grid. Finally, the molecular docking of the compounds was performed using the rigid ligand docking option with default parameters and the docking poses and predicted results were generated, as detailed previously [36].

### 2.3. Protein–Ligand Complex Analysis

Discovery Studio (https://www.3ds.com/products-services/biovia/, accessed on 4 December 2022) was exploited for binding pose analysis and the illustrations of two-dimensional ligand-receptor interactions were prepared. The binding analysis of a two-dimensional interpretation of interacting residues and interfaces with high-quality protein and ligand contacts was generated in BIOVIA (https://www.3ds.com/products-services/biovia/, accessed on 4 December 2022) [37]. The result was an instructive depiction of the intermolecular interactions and their strengths, which included hydrogen bonds, hydrophobic contacts, and atom accessibilities.

### 2.4. Binding Energy and Dissociation Constant

The dock score is the score obtained directly from the docking software Dock v. 6.9 used in this study and demonstrates how fit the ligand is in the binding site. In addition to the dock score, the binding energy and dissociation constant terms were also evaluated for the ligand–protein complexes using another software, X-Score v. 1.2.11 [38].

### 2.5. Binding-Pose Comparison Analyses

The binding poses of the organotin compounds were compared to that of the native ligands in order to check if the docked organotin ligands were bound to the same ligand binding sites of AR, ERα, and ERβ where the respective native ligand were bound. The 3D structure of AR, ERα, and ERβ proteins had bound ligands (native) whose binding pocket ensured that the docking site chosen was unerring. Further, within the binding site, the interacting residues common for the organotin compounds and the native ligand were compared.

### 2.6. Molecular Dynamics (MD) Simulation Analysis

MD simulation was carried out with the Schrodinger Desmond tool [39] to obtain an insight into the binding stability of the docked complex of AR and TBT. Prior to MD simulation, docked complex structures were minimized using the protein preparation wizard of Schrodinger where the hydrogen bond network was optimized at pH 7.4, and final restrained minimization was performed using the OPLS3e force field [39]. Further minimized structures were incorporated within an orthorhombic box solvated with a TIP3P water model using a system builder module, and then 0.15 M NaCl counter ions were added to neutralize the system. All prepared systems were relaxed before the MD simulation by a series of energy minimization and short MD simulations. Finally, the MD simulations were subjected to a 100 ns time period for individual systems, and the coordinates were saved at an interval of 50 ps at 300 K temperature with 1.0325 bar pressure. The simulation event analysis module in Desmond was utilized further to analyze the simulation results. The dynamic profile of the AR/TBT complex was assessed by root mean square deviation (RMSD) from the 100 ns trajectory. Root mean square fluctuation, i.e., protein residue fluctuation, of the AR/TBT complex was monitored. In addition, a radius of gyration (Rg) calculation over MD simulation was performed, which is a major indication of structural compactness. Finally, an estimation of the total change in solvent accessibility surface area (SASA) of AR/TBT complex simulation was performed. The method has been detailed previously [40,41].

## 3. Results

### 3.1. Molecular Docking Analyses

**Figure 2:**
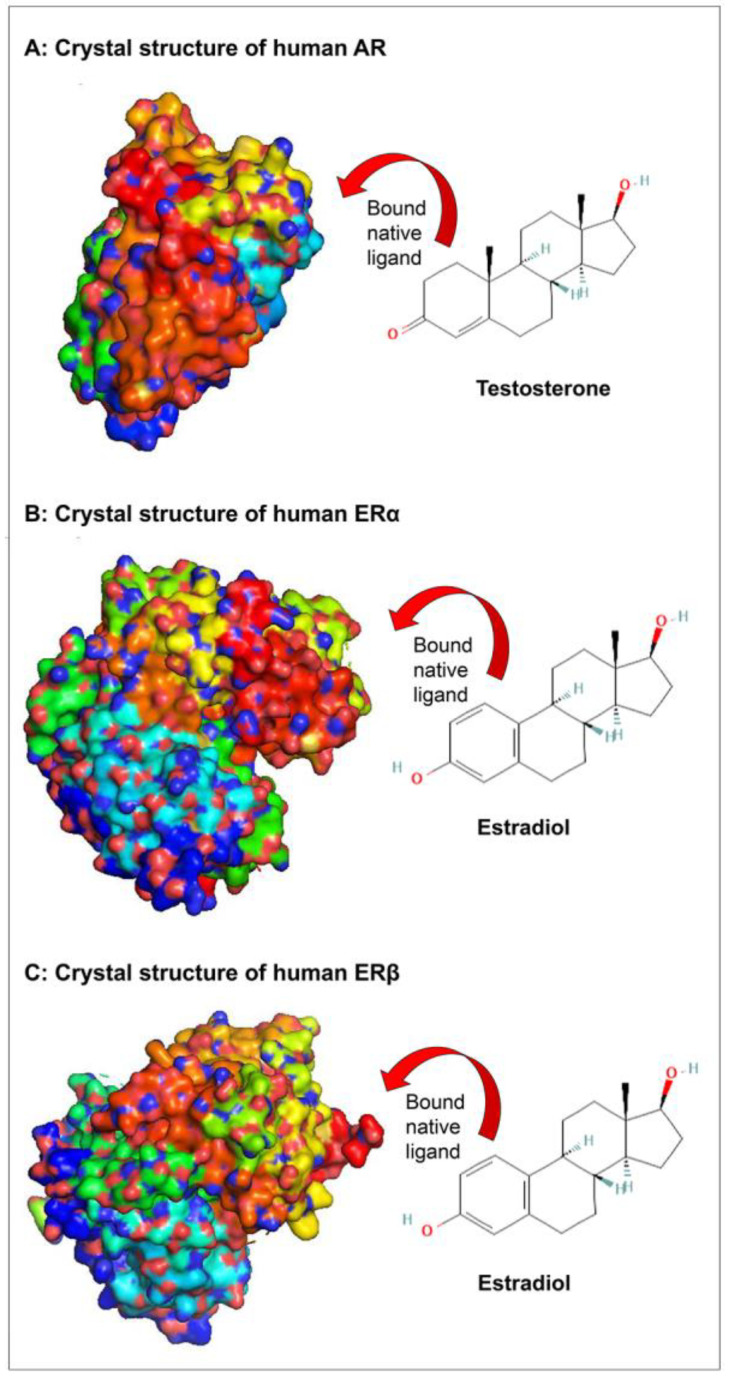
Surface representation of crystal structure of sex steroid hormone receptors containing the bound native ligands in the respective binding sites. Panel (**A**): crystal structure of human androgen receptor (AR) ligand binding domain in complex with testosterone; Panel (**B**): crystal structure of human estrogen receptor α (ERα) ligand binding domain in complex with estradiol; and Panel (**C**): crystal structure of human estrogen receptor β (ERβ) ligand binding domain in complex with estradiol.

The crystal structures of sex steroid hormone receptors, AR, ERα, and ERβ, containing the bound native ligands in the respective binding sites are presented in Figure 2. The analysis of binding strength scores of the organotins, i.e., TBT, DBT, MBT, TPT, DPT, MPT, and ACT in the present study with sex steroid receptors, AR, ERα, and ERβ and the interaction analysis of the ligand–protein complexes showed that four of seven organotin ligands bound well within the binding sites of the receptors (Table 1). The exception was MBT, which showed a negative but low value of dock score for all the three receptors, indicating a weak binding. Further analysis for other parameters for this organotin was not performed. In addition, TPT showed a high positive value of dock score for all the three receptors, indicating that it did bind to the receptors (a good binding is indicated by a higher negative dock score) and, lastly, ACT somehow could not be docked even after varying the default parameters. Therefore, MBT, TPT, and ACT were not considered for molecular interactions and binding pose analysis with the three receptors. Only four organotins, i.e., TBT, DBT, DPT, and MPT, were included in further analysis.

### 3.2. Molecular Docking of Organotin Compounds with AR

**Figure 3:**
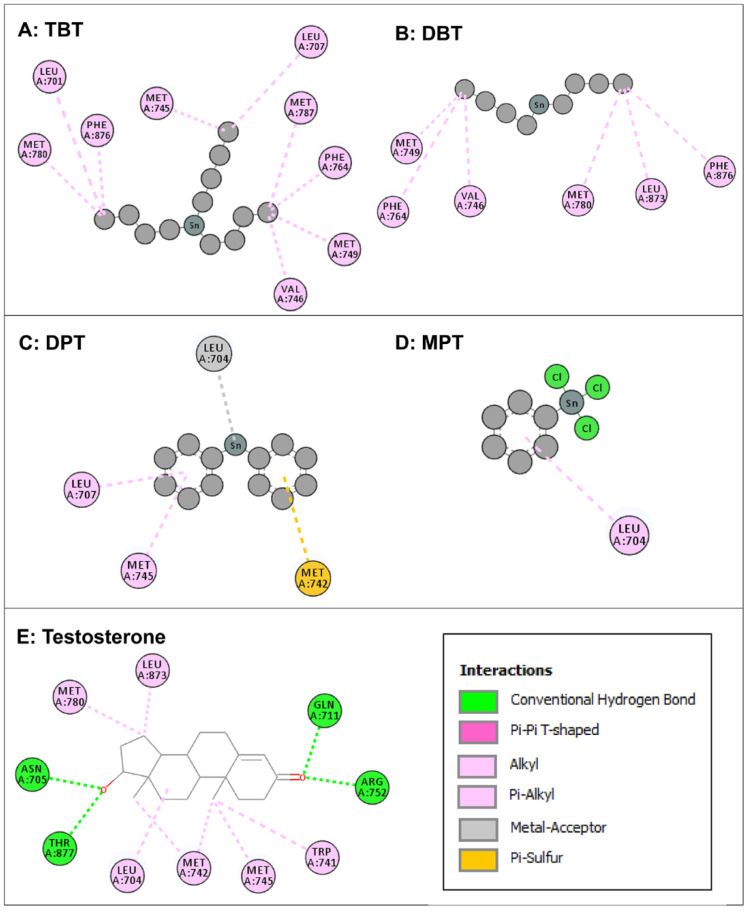
Two-dimensional interaction maps of amino acid residues of androgen receptor (AR) interacting with four organotin ligands during molecular docking in the ligand-binding site of AR. Amino acid residue interactions of the bound native ligand of AR (testosterone) are also shown. Tributyltin (TBT, Panel **A**); dibutyltin (DBT, Panel **B**); diphenyltin (DPT; Panel **C**); monophenyltin (MPT, Panel **D**); and testosterone (Panel **E**). Amino acid residues with alkyl or pi-alkyl bonds are indicated in light pink color, an amino acid with pi-sulfur bond is indicated in yellow color, and an amino acid with a metal acceptor bond is indicated in grey color. Interactions box: the depicted colors for interaction legends apply to all five interaction maps in the figure.

Intermolecular interactions of four organotin ligands, i.e., TBT, DBT, DPT, and MPT in complex with AR were analyzed and identified. All docked ligands were found to have similar binding poses to the native ligand, thus providing credence to the docking accuracy. All the organotin ligands bound well within the binding site of AR. The values for the dock score, binding energy, and dissociation constant were comparable among the ligands and were lower but close to native ligand, testosterone, indicating tight binding and similar binding strength to AR (Table 1). The two-dimensional interaction maps of amino acid residues of AR interacting with four organotin ligands and native bound ligand, testosterone, are illustrated in Figure 3.

The amino-acid residue interactions in the docking poses of organotin ligands, TBT, DBT, DPT, and MPT with AR are presented (Figure 3A–D). The docking pose analysis revealed that TBT interacted with nine amino acid residues of AR, i.e., Leu-701, Leu-707, Met-745, Val-746, Met-749, Phe-764, Met-780, Met-787, and Phe-876, and DBT interacted with six amino-acid residues of AR, i.e., Val-746, Met-749, Phe-764, Met-780, Leu-873, and Phe-876. In addition, DPT interacted with four amino acid residues of AR, i.e., Leu-704, Leu-707, Met-742, and Met-745, whereas MPT interacted with one amino acid residue (Leu-704) of AR. These residues were significantly important for the AR/organotin ligand bonding interaction in the active sites. The true conformation of bound native ligand testosterone in the binding site of AR is shown (Figure 2A), and the interacting amino acid residues are illustrated (Figure 3E). For testosterone, 10 amino acid residues were interacting with AR, i.e., Leu-704, Asn-705, Gln-711, Trp-741, Met-742, Met-745, Arg-752, Met-780, Leu-873, and Thr-877. Four hydrogen bonds were formed by Asn-705, Gln-711, Arg-752, and Thr-877, in addition to various other molecular interactions. When the interacting amino acid residues of AR for each of the four organotins were compared with those for native ligand (testosterone), two of nine interacting residues (Met-745, Met-780) for TBT, two of six interacting residues for DBT, three of four interacting residues for DPT, and the single interacting residue for MPT, were common between indicated organotin ligands and testosterone (Table 2). This suggested that the organotin ligands are bound in the same ligand binding pocket as the native ligand. Thus, on a preliminary basis, organotins have the potential to interfere with the binding of testosterone to AR and act as interfering compounds for androgen signaling.

### 3.3. Molecular Docking of Organotin Compounds with ERα

**Figure 4:**
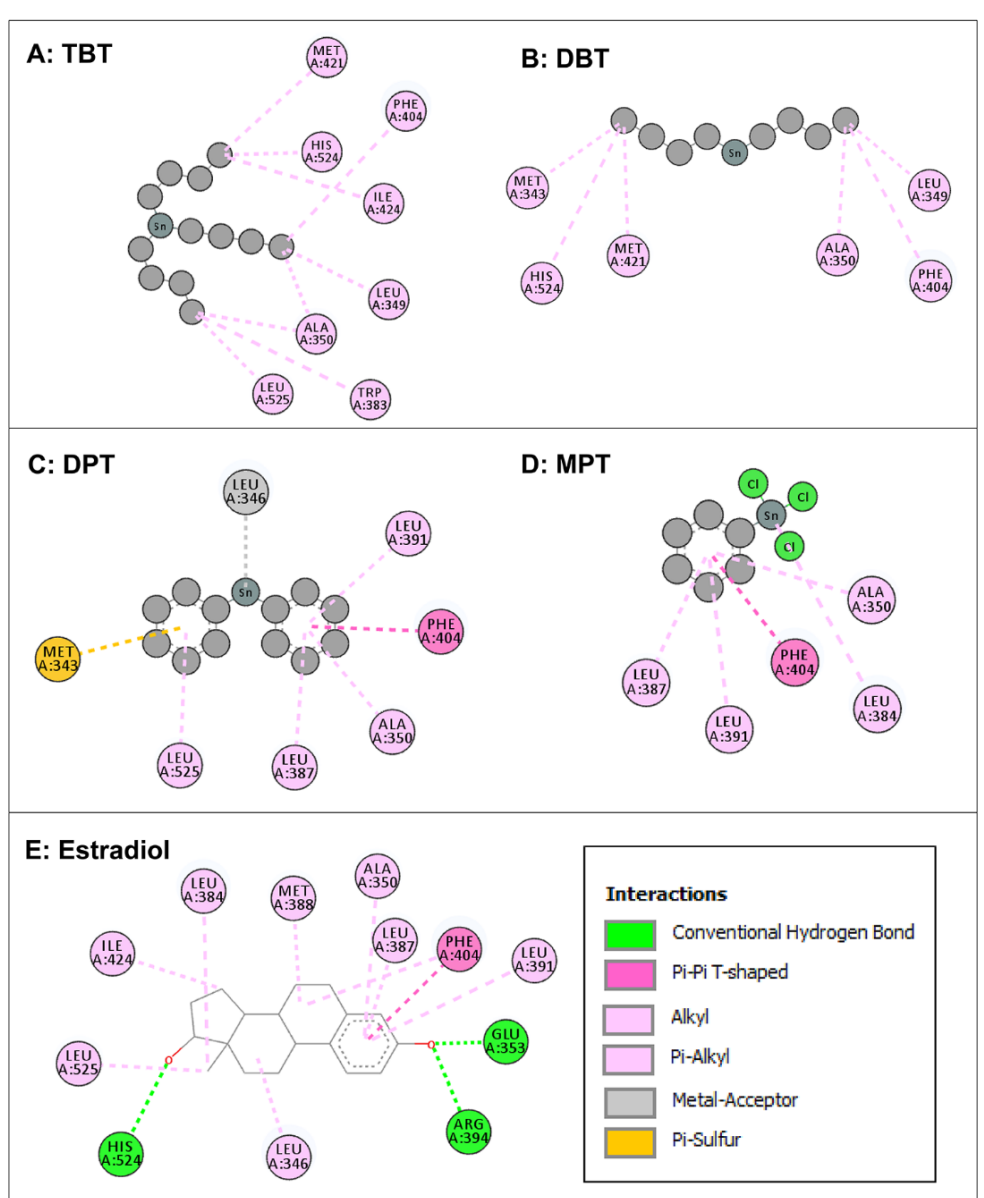
Two-dimensional interaction maps of amino acid residues of estrogen receptor (ERα) interacting with four organotin ligands during molecular docking in the ligand-binding site of ERα. Amino acid residue interactions of the bound native ligand of ERα (estradiol) are also shown. Tributyltin (TBT, Panel **A**); dibutyltin (DBT, Panel **B**); diphenyltin (DPT; Panel **C**); monophenyltin (MPT, Panel **D**); and estradiol (Panel **E**). Amino acid residues with alkyl or pi-alkyl bonds are indicated in light pink color, an amino acid with pi-sulfur bond is indicated in yellow color, amino acids with pi-pi T-shaped bond are indicated in dark pink color, and an amino acid with a metal acceptor bond is indicated in grey color. Interactions box: the depicted colors for interaction legends apply to all five interaction maps in the figure.

Intermolecular interactions of four organotin ligands, i.e., TBT, DBT, DPT, and MPT in complex with ERα were analyzed and identified. All docked ligands were found to have similar binding poses to the native ligand, thus providing credence to the docking accuracy. All the organotin ligands bound well within the binding site of ERα. The values for the dock score, binding energy, and dissociation constant were comparable among the ligands and were lower but close to native ligand, estradiol, indicating tight binding and similar binding strength to ERα (Table 1). The two-dimensional interaction maps of amino acid residues of ERα interacting with four organotin ligands and native bound ligand, estradiol, are illustrated in Figure 4.

The amino-acid residue interactions in the docking poses of organotin ligands, TBT, DBT, DPT, and MPT with ERα are presented (Figure 4A–D). The docking pose analysis revealed that TBT interacted with eight amino acid residues of ERα, i.e., Leu-349, Ala-350, Trp-383, Phe-404, Met-421, Ile-424, His-524, and Leu-525, and DBT interacted with six amino-acid residues of ERα, i.e., Met-343, Leu-349, Ala-350, Phe-404, Met-421, and His-524. In addition, DPT interacted with seven amino acid residues of ERα, i.e., Met-343, Leu-346, Ala-350, Leu-387, Leu-391, Phe-404, and Leu-525, whereas MPT interacted with five amino acid residues of ERα, i.e., Ala-350, Leu-384, Leu-387, Leu-391, and Phe-404. These residues were significantly important for the ERα/organotin ligand bonding interaction in the active sites. The true conformation of the bound native ligand estradiol in the binding site of ERα is shown (Figure 2B), and the interacting amino acid residues are illustrated (Figure 4E). For estradiol, 12 amino acid residues were interacting with ERα, i.e., Leu-346, Ala-350, Glu-353, Leu-384, Leu-38&, Met-388, Leu-391, Arg-394, Phe-404, Ile-424, His-524, and Leu-525. Three hydrogen bonds were formed by Arg-394, Gln-353, and His-524, in addition to various other molecular interactions. When the interacting amino acid residues of ERα for each of the four organotins were compared with those for bound native ligand (estradiol), five of eight (62%) interacting residues for TBT, three of six (50%) interacting residues for DBT, six of seven (86%) interacting residues for DPT, and the five of five (100%) interacting residue for MPT, were common between indicated organotin ligands and estradiol (Table 3). This suggested that the indicated four organotin ligands potentially bind in the same binding pocket of ERα as the native ligand estradiol. Thus, these compounds may interfere with the binding of estradiol to its receptor and cause dysfunction of estradiol signaling.

### 3.4. Molecular Docking of Organotin Compounds with ERβ

**Figure 5:**
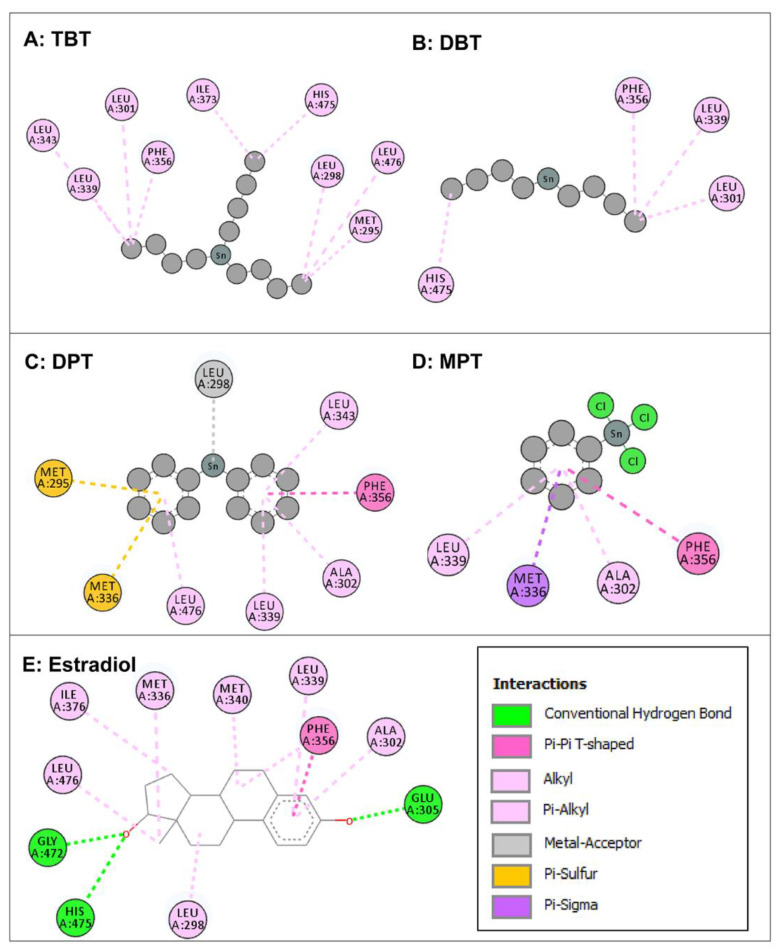
Two-dimensional interaction maps of amino acid residues of estrogen receptor β (ERβ) interacting with four organotin ligands during molecular docking in the ligand-binding site of ERβ. Amino acid residue interactions of the bound native ligand of ERβ (estradiol) are also shown. Tributyltin (TBT, Panel **A**); dibutyltin (DBT, Panel **B**); diphenyltin (DPT; Panel **C**); monophenyltin (MPT, Panel **D**); and estradiol (Panel **E**). Amino acid residues with alkyl or pi-alkyl bonds are indicated in light pink color, an amino acid with pi-sulfur bond is indicated in yellow color, amino acids with pi-pi T-shaped bonds are indicated in dark pink color, an amino acid with a metal acceptor bond is indicated in grey color, and an amino acid with pi-sigma bond is indicated in purple color. Interactions box: the depicted colors for interaction legends apply to all five interaction maps in the figure.

Intermolecular interactions of four organotin ligands, i.e., TBT, DBT, DPT, and MPT in complex with ERβ were analyzed and identified. All docked ligands were found to have similar binding poses to the native ligand, thus providing credence to the docking accuracy. All the organotin ligands bound well within the binding site of ERβ. The values for the dock score, binding energy, and dissociation constant were comparable among the ligands and were lower but close to native ligand, estradiol, indicating tight binding and similar binding strength to ERβ (Table 1). The two-dimensional interaction maps of interacting amino acid residues of ERβ with four organotin ligands and native bound ligand, estradiol, are illustrated in Figure 5.

The amino-acid residue interactions in the docking poses of organotin ligands, TBT, DBT, DPT, and MPT with ERβ are presented (Figure 5A–D). The docking pose analysis revealed that TBT interacted with eight amino acid residues of ERβ, i.e., Met-295, Leu-298, Leu-301, Leu-339, Leu-343, Phe-356, Ile-373, His-475, and Leu-476, and DBT interacted with four amino-acid residues of ERβ, i.e., Leu-301, Leu-339, Phe-356, and His-475. In addition, DPT interacted with eight amino acid residues of ERβ, i.e., Met-295, Leu-298, Ala-302, Met-336, Leu-339, Leu-343, Phe-356, and Leu-476, whereas MPT interacted with four amino acid residues of ERβ, i.e., Ala-302, Met-336, Leu-339, and Phe-356. These residues were significantly important for the ERβ/organotin ligand bonding interactions in the active sites. The true conformation of the bound native ligand estradiol in the binding site of ERβ is shown (Figure 2C), and the interacting amino acid residues are illustrated (Figure 5E). For estradiol, 11 amino acids were interacting with ERβ, i.e., Leu-298, Ala-302, Glu-305, Met-336, Leu-339, Met-340, Phe-356, Ile-376, Gly-472, His-475, and Leu-476. Three hydrogen bonds were formed by Gln-305, Gly-472, and His-475, in addition to various other molecular interactions. When the interacting amino acid residues of ERβ for each of the four organotins were compared with those for bound native ligand (estradiol), five of nine (56%) interacting residues for TBT, three of four (75%) interacting residues for DBT, six of eight (75%) interacting residues for DPT, and the four of four (100%) interacting residue for MPT, were common between indicated organotin ligands and estradiol (Table 4). This suggested that the indicated four organotin ligands potentially bind in the same binding pocket of ERβ as the bound native ligand estradiol. Thus, these compounds may interfere with the binding of estradiol to its receptor (ERβ) and cause dysfunction of estradiol signaling.

### 3.5. Molecular Dynamics (MD) Simulation

**Figure 6:**
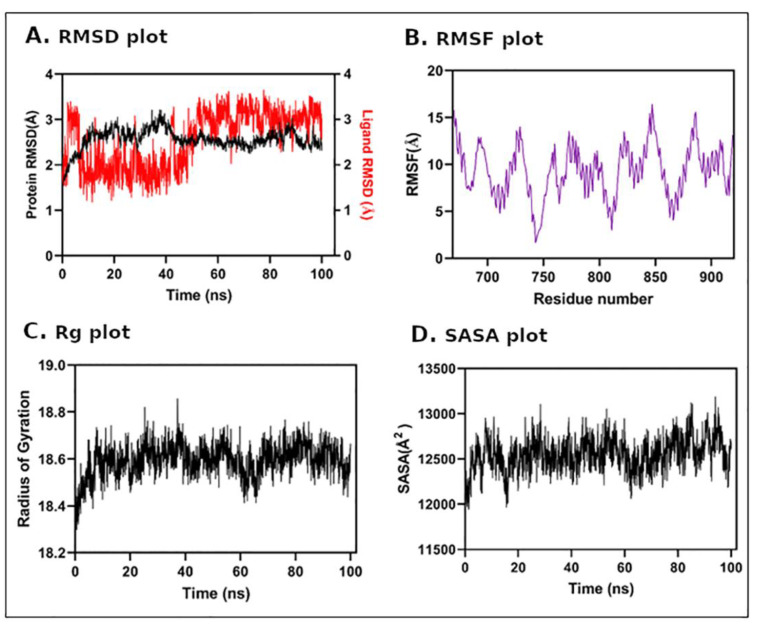
Molecular dynamics simulation analysis of androgen receptor in complex with tributyltin. Panel (**A**): root mean square deviation plot; Panel (**B**): root mean square fluctuation analysis; Panel (**C**): radius of gyration; and Panel (**D**): solvent accessible surface area.

In order to obtain an insight into protein–ligand complex stability, we performed an all-atomic molecular dynamics simulation of a 100 ns time period for AR and TBT complex. The dynamic profile of the complex was assessed by RMSD from the 100 ns trajectory. The results of the RMSD plot indicated that the AR/TBT ligand complex showed a stable interaction throughout MD simulation, and the system achieved equilibrium during the early period of MD simulation i.e., after 10 ns (Figure 6A). Both AR and TBT showed less deviation throughout MD simulation, which was 2.6 ± 0.22 and 2.6 ± 0.62, respectively. RMSF, i.e., protein residue fluctuation was also monitored during MD simulation (Figure 6B). Similarly, a Rg calculation over MD simulation, which is a major indication of structural compactness, was performed (Figure 6C). The Rg results showed that the AR/TBT complex maintains its compactness throughout and does not fluctuate much, and the average Rg was 19 ± 0.06 Å. Further, we estimated the total change in SASA of AR/TBT complex simulation (Figure 6D). The results showed that the SASA profile did not change during the whole simulation period indicating no huge structural deviation during MD simulation. Thus, the results indicate good stability of the AR/TBT protein–ligand complex.

## 4. Discussion

The objective of this study was to characterize the structural binding interactions of seven commonly available OTCs, viz., TBT, DBT, MBT, TPT, DPT, MPT, and ACT, against sex-steroid nuclear receptors, i.e., AR, ERα, and ERβ. As already indicated, docking analysis showed that four of seven organotin ligands, i.e., TBT, DBT, DPT, and MPT, bound well within the binding sites of the three receptors. The remaining three organotin ligands, MBT, TPT, and ACT, showed a weak binding or did not bind with the receptors and, hence, further analysis of these ligands for molecular interactions and binding pose analysis was not considered. The dock score, binding energy, and dissociation constants for the four organotins with AR, ERα, and ERβ indicated good docking and binding within the ligand binding sites of the receptors. Similar binding poses of the organotin ligands and the respective bound native ligands along with close similarity in binding energies indicated good binding and thus provided credence to the docking accuracy. In addition, high overlapping/commonality of interacting residues of each receptor for the docked ligands and respective native ligands provided further support for good docking. This was especially true for the interacting residues of ERα, and ERβ for the organotin ligands and native ligands as ERα, and ERβ showed better structural binding characteristics compared to AR. The good docking and stability of docking complex was further supported by the results of MD simulation of a representative organotin/receptor complex (TBT/AR), with a RMSD plot showing a stable interaction of TBT with AR with low deviation of ligand within good acceptable range. In addition, RMSF and Rg plots showed maintenance of compactness of the complex along with no change in the SASA profile during the whole simulation period. Thus, the MD simulation also provided support and indicated good stability of the receptor–ligand complex. Taken together, all the above results suggested that the organotin ligands are bound tightly in the same ligand binding pockets of AR, ERα, and ERβ as their respective bound native ligands, testosterone and estradiol, especially for the ERα, and ERβ. Thus, the suggested hypothesis that OTCs may interfere with the natural interaction between sex steroids and their receptors by binding to sex steroid nuclear receptors was supported. Hence, the indicated organotins have the potential to interfere with the binding of testosterone and estradiol17β to their respective receptors and result in dysfunction of steroid receptor signaling.

Previous reports on the in silico studies of organotins with sex steroid nuclear receptors are not available to the best of our knowledge. However, organotins, TBT and TPT, were shown to have competitive binding antagonistic activity against human ER in vitro [42]. The inhibitory effect on ER similar to other ER antagonists, such as 4-hydroxytamoxifen, was shown on ER-dependent reporter gene transcriptional activation by TBT and TPT at very low concentrations by interactions between human ERβ LBD and the co-activator SRC1 in a yeast two-hybrid detection system. In addition, TBT and TPT stimulated LA16 cells (that stably expressed androgen-responsive luciferase reporter gene and proliferates in response to androgen) and enhanced both AR-dependent transcription of luciferase gene and cell growth similar to dihydrotestosterone [43]. Simultaneous treatment of LA16 cells with dihydrotestosterone and TBT or TPT caused synergistic effects on AR activation, but an androgen antagonist, flutamide, did not inhibit the TBT- or TPT-induced AR activation, suggesting a novel mechanism other than the ligand-binding site of AR. TBT and TPT were also shown to interact with other nuclear receptors, e.g., as nanomolar agonists of retinoid X receptor (RXR) and peroxisome proliferator-activated receptor γ (PPARγ). In this regard, TBT and TPT exposure to a RXR-transfected human choriocarcinoma cell line (JEG-3 cells) stimulated luciferase expression, indicating activation of RXR [44]. In vivo and in vitro studies on organotins in marine invertebrates, fish, mammals, and laboratory animals have shown the profound endocrine disruption effects of organotins on reproductive and other functions. Development of imposex in mollusks even with exposure to very low TBT concentrations is the most sensitive and well known reproductive phenotypic effect of endocrine disruption [6]. The endocrine disruption by organotins in various species is believed to occur through three main mechanisms, i.e., (1) increased testosterone—organotins cause an androgen abundance by inhibiting the aromatase in females [45], (2) the APGWamide neuropeptide activation—organotins induce abnormally high levels of peptide APGWamide, which cause the development of male-like tissues subsequently producing androgens to promote the male-like sexual growth, and (3) the RXR agonism/activation—organotins cause abnormal activation of the RXR signaling pathway through ligand binding or by increasing the retinoid [23]. RXRs in association with other nuclear receptors, such as PPAR, etc., regulate cellular development and differentiation, metabolism, and cell death. In addition, recently inactivation of UDP-glucuronosyltransferases (UGTs) was also proposed to be another mechanism by which organotins can cause abnormal reproductive health effects; UGTs regulate metabolic inactivation of many endogenous hormones [46]. Inhibition of UGTs, including UGT2B15 (regulates catalysis of dihydrotestosterone glucuronidation) and UGT1A1 (regulates catalysis of estradiol-3-O-glucuronidation) by organotins such as TBT and TPT, may interfere with glucuronidation of endogenous sex hormones and thus inhibit their termination, resulting in abnormal endocrine actions [46]. The endocrine disruption mechanism of organotins in humans is not clear but has been postulated to most likely involve increased testosterone [4]. In addition, the potential for TBT and other organotins to act as obesogens in humans and to interfere with endocrine regulation of adipogenesis such as stimulation of preadipocyte differentiation into adipocytes in a PPARγ-dependent manner is also very concerning.

Very limited epidemiological studies have been reported on the association of organotin exposure and human reproductive problems. Several reports have mentioned general toxic effects associated with acute human organotin exposure, such as seizures, visual disturbances, paraparesis, forgetfulness, fatigue, weakness, loss of motivation, depression, and attacks of rage; some symptoms persist for at least three years [2,47,48]. Information associated with long term exposure is not reported. In other studies, severe toxicity of TPT showed neurotoxic symptoms, such as cerebellar syndrome, hearing impairment, and loss of consciousness with paroxysmal activity on electroencephalography [49]. In general population, significant levels of the organotins TBT and TPT have been detected in the blood of human male and female volunteers [50]. In another study, triorganotins, such as TBT and TPT, were detected in 37% and 99% of the placental tissues of women in Denmark and Finland, respectively, and a positive correlation between organotin (DBTCl) levels in the human placenta and incidence of cryptorchidism was reported in newborns in Denmark [21]. In addition, in the same study, the blood LH levels in four-month-old boys were negatively correlated, whereas the inhibin B levels were positively correlated with TBTCl in the placenta of women from Finland.

Several studies have reported adverse effects of organotins in male reproductive function in laboratory species [4,27]. In this regard, rat testes were reported to accumulate tin after three days of TBT exposure, resulting in reproductive abnormalities [25]. TBT induced weight gain and improved the food efficiency of male rats [51]. In another study [52], TPT resulted in decreased sperm count and motility in a dose-dependent manner, an impaired sperm histone–protamine replacement process, and significantly increased incidence of sperm deformities, as well as impaired proliferation of spermatogonia in adult male rats. In a recent study, TBT exposure decreased the number of Leydig cells and inhibited androgen production in rats [53]. In male mice, TBT exposure early after birth was associated with reduction in the weight of testis, epididymis, prostate, and seminal vesicles [54,55]. In zebra fish, TBT exposure during early development induced male bias and reduced or completely inhibited sperm motility, caused the absence of flagella, and resulted in only abnormal spermatozoa in semen in the exposed male offspring [56]. Similarly, exposure of ACT to adult male and female zebra fish altered the gene expression related to reproductive function, such as for LH in pituitary and for aromatase in gonads, along with reductions in estrogen in both sexes [57]. In addition, ACT exposure was associated with impaired spermatogenesis in males.

In addition to in vivo studies, in vitro studies have shown that TPT exerted inhibitory effects on the activity of human blood steroidogenic enzymes, i.e., 5-alpha-reductase type 2, cytochrome P450 aromatase, 17-beta-HSD type 3, 3-beta-HSD type 2, and 17-beta-HSD type 1 through the interaction with critical cysteine residues [50]. TBT, DBT and TPT salts were shown to inhibit human 5-alpha-reductase type 1 and 5-alpha-reductase type 2 enzymes, which are required for activation of androgens [58]. In vitro acute or short-term exposure of bovine sperm to TBT was associated with decrease in total motility, progressive motility, curvilinear velocity, and beat-cross frequency, along with a lower mitochondrial membrane potential [26]. In addition, TBT-exposed sperm resulted in a reduced cleavage rate and a lower rate of 8-16 cell morula development compared to embryos from unexposed sperms. DBT decreased the production of androgens from rat immature Leydig cells both under basal and LH stimulated conditions [59]. In another study [53], TBT exposure of immature rat Leydig cells in vitro reduced androgen production, cell viability, and cell cycle progression, while increasing reactive oxygen species (ROS) and apoptosis. Similarly, exposure of rat Leydig cells and Sertoli cells to TPT induced a significant decrease in the expression of steroidogenic and apoptotic indicators; similar exposure did not affect spermatogonia cells [52].

In female animals, organotin exposure was associated with toxicity of female reproductive system [3,27]. The most well known effect of many organotins, such as TBT and TPT, is the development of imposex in female gastropods, which refers to the development of male sexual organs in females, such as the penis and vas deferens [60,61,62]. In many laboratory mammalian animal models, TBT exposure was associated with reproductive, metabolic, and cardiovascular abnormalities, including hyperandrogenism, cystic ovarian follicles, irregular estrous cycle, elevated levels of LH, obesity, abnormal lipid profiles, glucose metabolism, and insulin resistance similar to those found in polycystic ovarian syndrome (PCOS) in women and animal models of PCOS [22,63,64,65]. In this regard, TBT exposure in female rats induced body weight gain and adiposity [66]. Exposure to TBT disrupts the proper functioning of the HPG axis of the female rats, probably in part through causing abnormal KISS and GnRH action, which regulate the reproductive axis of the hypothalamus and pituitary [64]. In this regard, TBT exposure in female rats caused irregular estrous cycles, downregulated hypothalamic GnRH mRNA expression, decreased exogenous KISS response, decreased basal and surge levels of LH, reduced exogenous GnRH responsiveness, decreased pituitary expression of both ERα and Erβ, increased testosterone, ovarian and uterine fibrosis, and decreased fertility. In mice and rats, TBT disrupts ovarian reserve, development of germ cells, folliculogenesis, steroidogenesis, ovulation, and CL formation [67]. In addition, in utero TBT exposure was associated with abnormal number and morphology of gonocytes with lipid droplets accumulating in the endoplasmic reticulum in the female rat offspring. In this regard, the placenta has been shown to accumulates the organotins, as exposure to TBT resulted in rat placental TBT levels that were five times higher than those found in maternal blood and ten times higher than those found in milk [68]. In other studies, TBT caused irregular estrous cycles, disturbed ovarian development, including increased presence of atretic and cystic follicles, fewer CLs, antral follicles and increased levels of atretic follicles, hyperandrogenism, high levels of serum LH, and decreased levels of serum sex hormone-binding globulin, in addition to an increase in the RXR/PPAR signaling pathway and other proteins that are involved in androgen biosynthesis [65,69]. Exposure to ACT in adult female zebra fish was associated with alterations in the reproductive related gene expression, reduced estrogen, and increased testosterone in females [57]. Increased accumulation of ACT in F1 eggs and embryonic abnormalities were also found after parental exposure.

In vitro studies have also demonstrated effects of organotin on female hypothalamic-gonadal-axis and ovarian cells similar to in vivo effects. TBT exposure of ovarian theca cells from five species (human, sheep, cow, pig, and mouse) affected cholesterol trafficking, luteinization, and steroidogenesis in all five species [70]. The effect was, in part, through modulation of RXR, as shown by RXR antagonist and RXRα knockdown. In human granulosa-like tumor cell line KGN, TBT exposure reduced the mRNA expression of aromatase and its activity by 30% compared to control cells [71]. In addition, exposure to higher TBT concentrations resulted in KGN cell death within 24 h, whereas lower TBT concentration promoted apoptosis. Exposure of TBT in bovine cumulus–oocyte complex cultures reduced estrogen and testosterone levels along with expression of aromatase and 3-beta-HSD mRNA [72]. In addition, TBT exposure inhibited LH stimulated estrogen synthesis in follicular granulosa cells. TBT and DBT acted as partial competitive inhibitors of aromatase enzyme in human placenta and showed inhibition of human 3-beta HSD type I activity [73].

Taken together, the results of our in silico structural interactions of organotin ligands with sex steroid receptors support the previously reported adverse effects, as discussed above, for in vivo and in vitro studies in human and laboratory animals. The morphophysiological, hormonal, and molecular impairments discussed support our suggested hypothesis of perturbation of natural interaction between native ligands and sex steroid nuclear receptors leading to impaired reproductive function.

## 5. Conclusions

In the present study, molecular docking simulations of seven OTCs, viz., TBT, DBT, MBT, TPT, DPT, MPT, and ACT, against sex-steroid nuclear receptors, i.e., AR, ERα, and Erβ, were performed. The docking results showed that TBT, DBT, DPT, and MPT bound well within the binding sites of the three receptors. The remaining three organotin ligands, MBT, TPT, and ACT, did not bind with the receptors. All the indicated four organotin compounds interacted with each of the sex steroid receptors and bound deep into their ligand-binding sites, showing good dock score, binding energy, and dissociation constants that were comparable to the bound native ligands, testosterone, and estradiol. The good docking and stability of docking complex was also shown by MD simulation of organotin/receptor complex, with RMSD, RMSF, Rg, and SASA plots showing stable interaction, low deviation, and compactness of the organotin/receptor complex during the whole simulation period. In addition, high commonality of interacting residues of each receptor for the docked ligands and respective bound native ligands, especially for ERα and Erβ, which indicated that the organotin compounds bound tightly in the same ligand binding site of the receptor as the bound native ligand. To conclude, the study suggested that the indicated organotin compounds may interfere with the natural interaction between sex steroids and their receptors and cause dysfunction of steroid receptor signaling.

## Figures and Tables

**Table 1:** The binding strength scores, i.e., dock score, binding energy (BE), and dissociation constant (p*K***_d_**) for organotin compounds with androgen receptor, estrogen receptor α, and estrogen receptor β. The BE and p*K***_d_** values were calculated using X-Score. Lower (more negative) BE or dock score and higher p*K***_d_** denote better docking. Monobutyltin showed a weak binding (low dock score), and further analysis was not performed for its any other parameters. Triphenyltin did not bind to any of the receptors (high positive dock score), and azocyclotin could not be docked even after varying the default parameters.

Organotin Compounds	Androgen Receptor			Estrogen Receptor α			Estrogen Receptor β		
	Dock Score	BE (Kcal/mol)	p*K*_d_	Dock Score	BE (Kcal/mol)	p*K*_d_	Dock Score	BE (Kcal/mol)	p*K*_d_
Tributyltin	−30.18	−7.82	5.73	−31.26	−7.67	5.62	−30.80	−7.69	5.64
Dibutyltin	−27.79	−6.81	4.99	−26.79	−6.72	4.93	−27.68	−6.78	4.97
Diphenyltin	−28.40	−8.17	5.99	−27.64	−8.10	5.94	−22.76	−8.13	5.96
Monophenyltin	−23.93	−7.60	5.57	−25.67	−7.63	5.59	−24.75	−7.66	5.61
Native ligand	-	−10.36	7.60	-	−9.78	7.17	-	−9.95	7.29
Monobutyltin	−16.96	No analysis was performed due to low dock score		−16.94	No analysis was performed due to low dock score		−16.67	No analysis was performed due to low dock score	
Triphenyltin	772.83	No binding to receptor (dock score high positive)		122.80	No binding to receptor (dock score high positive)		176.40	No binding to receptor (dock score high positive)	
Azocyclotin	Could not be docked even after varying the default parameters.								

**Table 2:** Comparative list of amino acid residues of androgen receptor interacting with bound native ligand (testosterone), tributyltin, dibutyltin, diphenyltin, and monophenyltin. Residues within a row among native ligand and organotin ligands are overlapping interacting residues for the compounds.

Native (Testosterone)	Tributyltin	Dibutyltin	Diphenyltin	Monophenyltin
-	Leu-701	-	-	-
Leu-704	-	-	Leu-704	Leu-704
Asn-705	-	-	-	-
-	Leu-707	-	Leu-707	-
Gln-711	-	-	-	-
Trp-741	-	-	-	-
Met-742	-	-	Met-742	-
Met-745	Met-745	-	Met-745	-
-	Val-746	Val-746	-	-
-	Val-749	Met-749	-	-
Arg-752	-	-	-	-
-	Phe-764	Phe-764	-	-
Met-780	Met-780	Met-780	-	-
	Met-787			
Leu-873	-	Leu-873	-	-
-	Phe-876	Phe-876	-	-
Thr-877	-	-	-	-

**Table 3:** Comparative list of amino acid residues of estrogen receptor α interacting with bound native ligand (estradiol), tributyltin, dibutyltin, diphenyltin, and monophenyltin. Residues within a row among native ligand and organotin ligands are overlapping interacting residues for the compounds.

Native (Estradiol)	Tributyltin	Dibutyltin	Diphenyltin	Monophenyltin
-	-	Met-343	Met-343	-
Leu-346	-	-	Leu-346	-
-	Leu-349	Leu-349	-	-
Ala-350	Ala-350	Ala-350	Ala-350	Ala-350
Glu-353	-	-	-	-
Leu-384	-	-	-	Leu-384
-	Trp-383	-	-	-
Leu-387	-	-	Leu-387	Leu-387
Met-388	-	-	-	-
Leu-391	-	-	Leu-391	Leu-391
Arg-394	-	-	-	-
Phe-404	Phe-404	Phe-404	Phe-404	Phe-404
-	Met-421	Met-421	-	-
Ile-424	Ile-424	-	-	-
His-524	His-524	His-524	-	-
Leu-525	Leu-525	-	Leu-525	-

**Table 4:** Comparative list of amino acid residues of estrogen receptor β interacting with bound native ligand (estradiol), tributyltin, dibutyltin, diphenyltin, and monophenyltin. Residues within a row among native ligand and organotin ligands are overlapping interacting residues for the compounds.

Native (Estradiol)	Tributyltin	Dibutyltin	Diphenyltin	Monophenyltin
-	Met-295	-	Met-295	-
Leu-298	Leu-298	-	Leu-298	-
-	Leu-301	Leu-301	-	-
Ala-302	-	-	Ala-302	Ala-302
Glu-305	-	-	-	-
Met-336	-	-	Met-336	Met-336
Leu-339	Leu-339	Leu-339	Leu-339	Leu-339
Met-340	-	-	-	-
-	Leu-343	-	Leu-343	-
Phe-356	Phe-356	Phe-356	Phe-356	Phe-356
-	Ile-373	-	-	-
Ile-376		-	-	-
Gly-472	-	-	-	-
His-475	His-475	His-475	-	-
Leu-476	Leu-476	-	Leu-476	-

## Data Availability

The majority of the data for the results of this study are provided in the main manuscript. In addition, any specific data are available from the corresponding author.
